# Measuring e-Professional Behavior of Doctors of Medicine and Dental Medicine on Social Networking Sites: Indexes Construction With Formative Indicators

**DOI:** 10.2196/50156

**Published:** 2024-02-27

**Authors:** Marko Marelić, Ksenija Klasnić, Tea Vukušić Rukavina

**Affiliations:** 1 Andrija Štampar School of Public Health School of Medicine University of Zagreb Zagreb Croatia; 2 Department of Sociology Faculty of Humanities and Social Sciences University of Zagreb Zagreb Croatia; 3 Biomedical Research Center Šalata School of Medicine University of Zagreb Zagreb Croatia

**Keywords:** e-professionalism, social media, formative index, social networking, doctors, medical, dental medicine

## Abstract

**Background:**

Previous studies have predominantly measured e-professionalism through perceptions or attitudes, yet there exists no validated measure specifically targeting the actual behaviors of health care professionals (HCPs) in this realm. This study addresses this gap by constructing a normative framework, drawing from 3 primary sources to define e-professional behavior across 6 domains. Four domains pertain to the dangers of social networking sites (SNSs), encompassing confidentiality, privacy, patient interaction, and equitable resource allocation. Meanwhile, 2 domains focus on the opportunities of SNSs, namely, the proactive dissemination of public health information and maintaining scientific integrity.

**Objective:**

This study aims to develop and validate 2 new measures assessing the e-professional behavior of doctors of medicine (MDs) and doctors of dental medicine (DMDs), focusing on both the dangers and opportunities associated with SNSs.

**Methods:**

The study used a purposive sample of MDs and DMDs in Croatia who were users of at least one SNS. Data collection took place in 2021 through an online survey. Validation of both indexes used a formative approach, which involved a 5-step methodology: content specification, indicators definition with instructions for item coding and index construction, indicators collinearity check using the variance inflation factor (VIF), external validity test using multiple indicators multiple causes (MIMIC) model, and external validity test by checking the relationships of the indexes with the scale of attitude toward SNSs using Pearson correlation coefficients.

**Results:**

A total of 753 responses were included in the analysis. The first e-professionalism index, assessing the dangers associated with SNSs, comprises 14 items. During the indicators collinearity check, all indicators displayed acceptable VIF values below 2.5. The MIMIC model showed good fit (χ^2^_13_=9.4, *P*=.742; χ^2^/df=0.723; root-mean-square error of approximation<.001; goodness-of-fit index=0.998; comparative fit index=1.000). The external validity of the index is supported by a statistically significant negative correlation with the scale measuring attitudes toward SNSs (r=–0.225, *P*<.001). Following the removal of 1 item, the second e-professionalism index, focusing on the opportunities associated with SNSs, comprises 5 items. During the indicators collinearity check, all indicators exhibited acceptable VIF values below 2.5. Additionally, the MIMIC model demonstrated a good fit (χ^2^_4_=2.5, *P*=.718; χ^2^/df=0.637; root-mean-square error of approximation<0.001; goodness-of-fit index=0.999; comparative fit index=1.000). The external validity of the index is supported by a statistically significant positive correlation with the scale of attitude toward SNSs (r=0.338; *P*<.001).

**Conclusions:**

Following the validation process, the instrument designed for gauging the e-professional behavior of MDs and DMDs consists of 19 items, which contribute to the formation of 2 distinct indexes: the e-professionalism index, focusing on the dangers associated with SNSs, comprising 14 items, and the e-professionalism index, highlighting the opportunities offered by SNSs, consisting of 5 items. These indexes serve as valid measures of the e-professional behavior of MDs and DMDs, with the potential for further refinement to encompass emerging forms of unprofessional behavior that may arise over time.

## Introduction

### Background

The development of social networking sites (SNSs) as a new form of media and communication channel has brought many changes to the health care system [1]. The widespread use of SNSs affects what we perceive as the professional behavior of health care professionals (HCPs) [2].

The rise in SNS users has sparked a growing interest in comprehending e-professionalism, particularly concerning SNSs. This specific facet of e-professionalism is becoming increasingly important. Over the past few years, numerous studies on the e-professionalism of HCPs have emerged [3,4], indicating a sustained momentum in generating scientific insights into e-professionalism.

### Defining and Measuring e-Professionalism

The American Board of Internal Medicine (ABIM) guidelines on medical professionalism define 3 fundamental principles and a set of 10 professional responsibilities (or commitments). Fundamental principles are the importance of patient welfare, the principle of patient autonomy, and the principle of social justice. Professional responsibilities are commitments to professional competence, honesty with patients, patient confidentiality, maintaining appropriate relations with patients, improving the quality of care, improving access to care, a just distribution of finite resources, scientific knowledge, maintaining trust by managing conflicts of interest, and commitment to professional responsibilities [5].

E-professionalism is a specific type of professionalism. Cain and Romanelli [6] defined e-professionalism as the attitudes and behaviors (some of which may occur in private settings) reflecting traditional professionalism paradigms that are manifested through digital media.

A large number of previous research around e-professionalism measured the perception of e-professionalism [7-11] and attitude toward e-professionalism [12-18]. Through cross-validation, Kelley et al [19] created an instrument for measuring professional behaviors in pharmacy students, and even though there are some thematic overlaps, it is not suitable for measuring online behavior.

E-professionalism is often defined as a value which justifies the operationalization that directs the measurement of professionalism toward the measure of attitude. Nevertheless, from the perspective of the professions themselves, although professionalism is taught and transferred through socialization into the profession as a value, for assessing the level of e-professionalism of doctors of medicine (MDs) and doctors of dental medicine (DMDs) the behavioral component is of greater interest. Professional behavior, rather than just attitude, constitutes a visible aspect of professionalism. It is through professional behavior that not only patients and colleagues perceive a doctor’s professionalism, but also it is subject to internal control according to Freidsonian principles [20], enabling the profession to enforce sanctions on the professional. Professionalism is a behavior rather than an attitude because it should not be a hypothetical or idealized concept, as Evans [21] writes, but should be perceived as a reality—an actual entity. However, it is a real entity only if it is operational. To be real, professionalism must be something that people—professionals—actually “do,” not just something that the government or any other agency wants them to do, or wrongly imagines them to be doing [21]. The disconnection between behavior and attitude is termed “cognitive dissonance” [22], a phenomenon already acknowledged as a threat to the e-professionalism of HCPs on SNSs [4].

The research focused on the medical and dental professions as the target populations. These 2 fields were chosen due to their fundamental similarities, enabling comparisons, as well as their differences, suggesting potential variations in e-professionalism. Both medical and dental professions are sociologically recognized as professions [20] and share the commonality of providing health services. This entails a significant patient-practitioner relationship in both disciplines. Comparing various health professions is a valuable approach, and existing literature has already established overlaps in core competencies [23].

The primary distinction driving the selection of these 2 professions is the orientation of MDs, particularly in the Croatian context, toward the public sector, whereas DMDs are oriented toward the private sector.

This paper seeks to develop a reliable and valid instrument for assessing the e-professional behavior of both MDs and DMDs.

### Normative Framework for Defining e-Professional Behavior

#### Overview

To define and measure e-professional behavior effectively, it is crucial to differentiate between professional and unprofessional behaviors. In our case, the primary objective of the normative framework is to delineate the content specifications, specifically the domains of instruments used to measure e-professional behavior.

The normative framework for assessing e-professionalism among MDs and DMDs draws upon 3 primary sources. While none of these sources alone is adequate for defining a comprehensive normative framework, each provides essential information crucial for its development. Some aspects of these sources overlap conceptually, while others offer unique insights necessary for crafting the framework.

The first source comprises the e-professional conduct guidelines established by the ABIM [5]. These guidelines, among the earliest to be published, were developed through an international collaboration involving multiple institutions. They address the fundamental principles of professionalism and outline the professional responsibilities expected of MDs.

The second source consists of guidelines aimed at fostering e-professional behavior among medical and dental students [24]. While initially targeted at this specific demographic, a significant portion of the recommendations is applicable to the e-professionalism of MDs and DMDs. Consequently, these guidelines serve as a valuable resource for “reconstructing” the components of a normative framework for e-professionalism. They aid in delineating acceptable and unacceptable behaviors on SNSs within the context of medical and dental professions.

The third source is Julie Skrabal’s research [9], where she used the grounded theory method to develop a theoretical framework for e-professionalism. Her study empirically demonstrated which behaviors on SNSs are perceived as unprofessional. While the research focused on nursing students, the identification of key domains and indicators comprising professional behavior on SNSs holds significant value and applicability to MDs, DMDs, and all HCPs.

Based on the analysis of these 3 sources, e-professionalism, or e-professional behavior, can be categorized into 6 domains. Four of these domains pertain to the dangers associated with SNSs: confidentiality, privacy, contact with patients, and fair distribution of resources. The remaining 2 domains concern the opportunities afforded by SNSs: proactive dissemination of information relevant to public health and maintaining scientific objectivity. Each of these 6 domains is elaborated upon below.

#### Confidentiality

Confidentiality encompasses behaviors that primarily contravene the Health Insurance Portability and Accountability Act (HIPAA) of 1996. It entails safeguarding patient confidentiality to ensure that information regarding the patient is not disclosed, even to the patient’s relatives, without the patient’s explicit consent.

Concerning behavior on SNSs, HIPAA violations predominantly involve the unauthorized publication of photos or confidential patient information [9]. Additionally, adopting fake names (pseudonyms) to share posts containing medical or dental information constitutes another unprofessional behavior [24].

#### Privacy

This domain pertains to profile privacy settings and the management of post visibility. Barlow et al [25] established a correlation between privacy settings and unprofessional behavior, particularly among medical students. Consequently, they recommended the adoption of “private visibility settings” to mitigate such behaviors. Monitoring privacy settings [24], controlling post visibility [9,24], and seeking permission before tagging colleagues in posts to safeguard their privacy [24] are advocated practices. Furthermore, it is advisable to refrain from publishing professionally inappropriate content on SNSs, including posts containing curses, vulgar expressions, inappropriate attire, or behavior [9,24].

#### Contact With Patients

This domain encompasses direct contact with patients via SNSs. Inappropriate expressions, political incorrectness, or derogatory remarks toward patients or any individual or group can severely tarnish the public’s perception of doctors’ professional conduct [24]. Additionally, using unofficial channels, such as SNSs, to communicate sensitive professional information is considered unprofessional behavior within this domain [9].

#### Fair Distribution of Resources

Fair distribution of resources, as acknowledged in the ABIM guidelines, is considered an essential aspect of professional responsibility. While the ABIM guidelines emphasize the avoidance of unnecessary interventions and examinations, resource distribution also extends to SNSs. Time, a valuable resource allocated by MDs and DMDs to their patients, is particularly relevant in this context. Derived from the fundamental principle of professionalism known as the “Principle of Social Justice,” striving for a fair distribution of health care resources is imperative [5]. Communication with patients via SNSs typically requires the doctor’s time, often during their free time since it is an informal communication channel. According to the principle of fairness, it would be considered unprofessional behavior if a doctor selectively chooses which patients they are willing to communicate with on SNS and which they are not.

#### Proactive Publication of Information of Public Health Interest

The dimension of proactive publication of professional information of public health interest is one of the recognized aspects of e-professionalism that highlights the opportunity aspect of using SNSs. These behaviors are not deemed unprofessional when avoided; however, they can significantly contribute to e-professionalism when practiced by MDs and DMDs. While Skrabal [9] emphasizes creating positive postings as the absence of criticism and negative comments, proactive posting as a deliberate action toward e-professionalism is acknowledged in another research [26].

#### Scientific Objectivity

Sharing knowledge on SNSs is indeed desirable and constitutes professional behavior. However, it is essential to clearly differentiate between personal or subjective medical opinions and scientifically based facts [24].

### Formative Approach in Measuring e-Professionalism

Most latent variables used in the social sciences are measured using reflective (effect) indicators [27,28]. According to a prevailing convention, indicators are seen as functions of the latent variable, whereby changes in the latent variable are reflected in changes in the observable indicators [27]. This is often true regarding constructs such as personality or attitude [28]. For example, attitude about SNSs affects respondents’ responses to the items posed to them. If someone has a negative attitude about SNSs, that attitude “guides” their responses. However, in the case where the direction of “influence” is reversed, and where the indicators are “causing” the latent variable instead of “being caused by it,” then we can talk about formative measures [28].

Index construction focuses on explaining the abstract (unobserved) variance, considers multicollinearity among indicators, and emphasizes the role of an indicator as a “predictor” (latent variable) rather than “a predicted variable” [27].

The choice of approach (reflexive vs formative) stems from the concept, that is, from the relationship between variables and constructs [29]. Jarvis et al [30] stated 4 conditions that can help discern whether a reflective or a formative model is appropriate: (1) the direction of causality between the construct and the indicator, (2) the interchangeability of the indicators, (3) covariance between indicators, and (4) the nomological network of construct indicators.

The first argument presented by Jarvis et al [30] is valid for our research because, unlike attitude, e-professional behavior stems from specific actions and decisions on SNSs. If someone refrains from posting pictures of patients, seeks permission from a colleague before mentioning them on SNS, actively controls the visibility of their posts, and takes similar actions, then these decisions contribute to their e-professional behavior.

For the second argument, e-professional behavior indicators are not interchangeable, even though they all measure e-professionalism. Posting a picture of a patient on an SNS is considered unprofessional behavior, but so is posting pictures from parties at work. Both behaviors are unprofessional, although they are not interchangeable in measurement (someone may frequently post photos of patients but rarely post workplace-related images).

The third argument states that covariance among indicators is unnecessary [30]. It is neither expected nor needed here because recognized behaviors within the normative framework can be entirely unrelated but still measure e-professional behavior (eg, sending a friend request to a patient and asking a colleague to mention them in a post).

The fourth argument suggests that the nomological network in the formative model can have different antecedents and consequences [30]. Indicators of e-professional behavior do not need to share the same antecedents because they can be driven by different motivations. A doctor may post pictures of patients because they believe it raises awareness about a particular illness (even though this act is unprofessional), while the motivation for unprofessional behavior, such as posting pictures from workplace parties, does not stem from the same motivation.

Based on these arguments, the behavioral component of e-professionalism measured in this paper conceptually corresponds to the formative approach.

We presume that other research in this area has not applied a formative approach in measuring e-professionalism because they have yet to define e-professionalism as a behavior.

Diamantopoulos and Winklhofer [28] proposed 4 key steps for validating indexes with formative indicators. The first step, content specification, refers to specifying the scope of the latent variable; in the second step, it is necessary to define the indicators; the third step refers to checking the collinearity of the indicators using the variance inflation factor (VIF) [28]. The fourth step is to assess the external validity of the index. Verification of the external validity of formative indices is often carried out by checking the relationship of the index with other measures and variables (as cited in [28]).

Although these 4 steps are sufficient for constructing and validating the index, it is possible to make an additional check of the external validity proposed by Diamantopoulos and Winklhofer [28]. This requires creating a model in which some reflective indicators are included (Diamantopoulos and Winklhofer [28] use 2) in the same model as the formative indicators. This model is called the multiple indicators multiple causes (MIMIC) model [28]. Acceptable overall model fit suggests retention of items in the formative model. If the exclusion of some items can significantly increase the model fit under the very strict condition that not a single exclusion would violate the content validity of the formative model, only then can the items be excluded.

In this paper, we have followed these 4 key steps for validating indexes with formative indicators. An additional step (the MIMIC model) was conducted before assessing the external validity of the index.

## Methods

### Sample

Quantitative survey data were collected using an online survey questionnaire. The Checklist for Reporting Results of Internet E-Surveys (CHERRIES) [31] is available in Multimedia Appendix 1. The required sample size was defined according to a conservative estimate often used for multivariate analyses, corresponding to a 10:1 ratio between the number of observations and the number of variables used in the questionnaire’s largest instrument [32]. In our case, that is a sample size of 280 (140 MDs and 140 DMDs). The type of sample was a nonprobabilistic purposive sample.

The study was a part of a long-term research project funded by the Croatian Science Foundation, UIP-05-2017 “Dangers and Benefits of Social Networks: E-Professionalism of Health Care Professionals – SMePROF” [33].

The mailing lists used to distribute the survey were the official full membership emailing lists of the Croatian Medical Chamber (CMC) and Croatian Chamber of Dental Medicine (CCDM). At the time of the survey, the CMC’s emailing list contained 15,562 email addresses of MDs, and the CCDM’s email list contained 7616 email addresses of DMDs. The email included a brief text about the study’s objective, the expected time to complete the survey, and the person and university responsible for conducting the study.

Participation in the survey was voluntary; there was no form of incentive to complete the survey. To ensure anonymity, no identification data were collected. Data were collected from February to July 2021, with 2 reminders sent in that period.

### Ethics Approval

Both the study and the questionnaire were approved by the ethical boards of the University of Zagreb School of Medicine (641-01/18-02/01) and the University of Zagreb School of Dental Medicine (05-PA-24-2/2018). In addition, formal approval was obtained from the governing bodies of both the CMC and CCDM for the use of the complete mailing lists of MDs and DMDs who are members of the CMC (900-06/20-01/11) and CCDM (900-01/21-01/02).

### Measures

The instrument for measuring the e-professional behavior of MDs and DMDs, presented in this study, is part of a more extensive questionnaire called SMePROF Project Survey Questionnaire on Social Media Usage, Attitudes, Ethical Values and E-professional Behaviour of Doctors of Medicine and Doctors of Dental Medicine, available at Viskić et al [34]. Although the questionnaire contained multiple instruments partially derived from previous studies [10,34,35], the instrument for measuring the e-professional behavior of MDs and DMDs is a novel instrument created by the authors. The instrument contains 20 items measured using the self-reporting approach, used to create 2 e-professionalism indexes, and the process is explained in the following parts of this paper. In validating indexes, an MIMIC model was used, which required 4 reflexive variables (y_1_-y_4_) measuring attitude toward e-professionalism. These items were taken from a validated instrument for measuring attitudes toward e-professionalism [35]. Descriptives of these 4 reflexive variables are shown in Multimedia Appendix 2. The MIMIC model was exclusively used as a method for validating the external validity of the indexes, and not for theory development.

The associations of indexes with theoretically related constructs were tested to assess the external validity. For this purpose, we used a validated instrument for measuring attitudes toward SNSs [36]. The instrument was translated into the Croatian language, and after additional reliability checks, 1 item was removed from the scale (“Potential and/or existing employers may use the information found on SNS to make decisions about prospective and/or existing employees”). The final instrument used had 12 items and Cronbach α=.70.

### Analytical Methods

A descriptive analysis of frequencies and percentage of responses was carried out, and distribution measures such as mean, range, SDs, and α_3_ measure of asymmetry were determined depending on appropriateness. Correlations between quantitative variables were tested with the Pearson correlation coefficient and phi coefficients of associations. The multicollinearity of the instruments was tested with the VIF. The MIMIC model was used to check the external validity of instruments with formative indicators. Data analysis was performed using IBM SPSS Statistics 26. IBM SPSS Amos 22 was used to test the MIMIC model.

## Results

### Survey Responses

A total of 1013 responses were collected. The response rate was 4.37% (1013/23,178). The final realized sample of the entire research contained the answers of 999 respondents, of which 75.4% (753/999) use at least one SNS, 67.3% (507/753) of the respondents were MDs and 32.7% (246/753) were DMDs. The sample was predominantly female (558/753, 74.1%) with an average age of 38 (SD 10.99) years. Most respondents worked in a public health institution (412/753, 54.7%), and the second most frequent type of workplace was a private institution with a contract with the Croatian Health Insurance Fund (CHIF; 148/753, 19.7%).

Previous research on the same sample [34] showed a significant difference in age, where MDs were older than DMDs with an average age of 39.26 years as opposed to 36.58 years, respectively, and in the type of employment, with more than two-thirds of DMDs (168/246, 68.2%) being employed in the private sector compared with only 20.5% (104/507) of MDs. All specialization status levels are included in the sample (Multimedia Appendix 3).

### The Construction of the e-Professionalism Index—The Danger Aspect of SNSs

Following the first step in creating the index, according to Diamantopoulos and Winklhofer [28], the content for the latent variable is specified below. In the second step, e-professional behaviors described in the normative framework were operationalized into an instrument for measuring the aspect of e-professionalism related to the dangers of SNSs (Table 1). The identified indicators are grouped into 4 domains: confidentiality, privacy, contact with patients, and fair distribution of resources. Items were evaluated on a frequency rating scale: 0=Never, 1=Rarely, 2=Occasionally, 3=Often; and the option “I have never been in a situation where this could happen” was added. It was essential to distinguish behaviors that could have happened but did not from those for which the respondent was not even in a situation to practice them. Depending on the direction and content of the items, the difference between the opportunity to behave in a certain way and the frequency of that behavior can mean the difference between professional and unprofessional behavior. In the case of items formulated in a positive direction (marked +), a higher frequency measures a higher level of e-professionalism. In the case of items formulated in a negative direction (marked –), higher frequency measures a lower level of e-professionalism.

**Table 1:** Domains, indicators, and items for the instrument of e-professionalism—the danger aspect of SNSs^a^.

Domain and indicator		Item	Direction^b^
**Confidentiality**			
	Disclosure of patient information.	I published some information about my patient.	–
	Publication of photographs of the patient without their consent.	I posted a photo of my patient without their knowledge.	–
	Hiding behind false names when posting online or anonymously posting medical information.	I shared medical/dental advice on SNS without my name being visible.	–
	Confidentiality of communication also applies to SNS.	I shared some information about the patient I received through SNS with others.	–
**Privacy of MDs and DMDs profiles**			
	Active management of the visibility of posts depending on their content.	Depending on the appropriateness of the content of my posts, I determine to whom they will be visible.	+
	Controlling the visibility of other people’s posts that include you, depending on their content.	If I notice that someone else has published something about me (eg, my picture, location, or similar), I control who will see it.	+
	Seeking prior approval from colleagues to publish information about them.	I asked a colleague’s permission to mention them in the post.	+
	Appropriate behavior on published content from a professional context.	I have posted content that shows informal situations at my workplace (eg, drinks with colleagues or parties at work).	–
	The use of profanity and other vulgar expressions in posts.	A curse word or some different vulgar expression occasionally slips out in my posts.	–
**Contact with patients**			
	Inappropriate expression in posts.	In my posts, I am cautious that my expression is entirely professional.	+
	Separation of professional and private communication.	I communicate with patients regarding medical/dental problems and treatment from a private profile.	–
	Inclusion of patient data obtained at SNS in the medical documentation without the patient’s consent.	I included information about the patient I found through SNS in the medical documentation without their knowledge.	–
	Sending a friend request to a patient or a patient’s family member.	Have you ever sent a “friend request“ to a patient or a member of the patient’s family from a private profile on an SNS?	–
**Fair distribution of resources**			
	Communication with patients via SNS and outside working hours is selective (the doctor chooses whom they respond to; patients without SNS cannot reach them)	On SNS, I choose which patients I will make contact with and which I will not.	–

^a^SNS: social networking site.

^b^For items formulated in a positive direction (marked +), a higher frequency measures a higher level of e-professionalism. In the case of items formulated in a negative direction (marked –), a higher frequency measures a lower level of e-professionalism.

The indicator “Sending a friend request to a patient or a member of the patient’s family” was not measured as frequency. Instead, the 4 offered answers were as follows: Yes, to the patient; Yes, to a family member; Yes, both; and No. The negative response is considered professional, while all other responses indicate unprofessional behavior.

The descriptive results for the items that measure the aspect of e-professionalism related to the dangers of SNSs are shown in Table 2. The items that measure e-professional behavior are marked with a “b.” All other items measure e-unprofessional behavior.

**Table 2:** E-professionalism (the dangers aspect of SNSs^a^) descriptives (N=753).

Danger aspects	Never, n (%)	Rarely, n (%)	Occasionally, n (%)	Often, n (%)	I have never been in a situation where this could happen, n (%)
1. I asked a colleague’s permission to mention them in the post.^b^	170 (22.6)	117 (15.5)	71 (9.4)	50 (6.6)^c^	345 (45.8)^c^
2. I shared some information about the patient that I received through SNS with other people.	368 (48.9)^c^	61 (8.1)	26 (3.5)	3 (0.4)	295 (39.2)^c^
3. I posted a photo of my patient without their knowledge.	492 (65.3)^c^	14 (1.9)	7 (0.9)	2 (0.3)	238 (31.6)^c^
4. I included information about the patient I found through SNS in the medical documentation without their knowledge.	484 (64.3)^c^	3 (0.4)	2 (0.3)	0 (0.0)	264 (35.1)^c^
5. I shared medical/dental advice on SNS without my name being visible.	503 (66.8)^c^	39 (5.2)	6 (0.8)	4 (0.5)	201 (26.7)^c^
6. Depending on the appropriateness of the content of my posts, I determine to whom they will be visible.^b^	295 (39.2)	99 (13.1)	93 (12.4)	71 (9.4)^c^	195 (25.9)^c^
7. If I notice that someone else has published something about me (eg, my picture, location, or similar), I control who will see it.^b^	209 (27.8)	106 (14.1)	104 (13.8)	177 (23.5)^c^	157 (20.8)^c^
8. I have published content that shows informal situations at my workplace (eg, drinks with colleagues or parties at work).	354 (47.0)^c^	181 (24.0)	84 (11.2)	17 (2.3)	114 (15.1)^c^
9. I published some information about my patient.	579 (76.9)^c^	22 (2.9)	5 (0.7)	2 (0.3)	145 (19.3)^c^
10. I communicate with patients regarding medical/dental problems and treatment from a private profile.	423 (56.2)^c^	133 (17.7)	64 (8.5)	14 (1.9)	119 (15.8)^c^
11. On SNS, I choose which patients I will make contact with and which I will not.	293 (38.9)^c^	74 (9.8)	65 (8.6)	76 (10.1)	245 (32.5)^c^
12. In my posts, I am cautious that my expression is entirely professional.^b^	51 (6.8)	61 (8.1)	111 (14.7)	366 (48.6)^c^	164 (21.8)^c^
13. A curse word or some other vulgar expression occasionally slips out in my posts.	494 (65.6)^c^	86 (11.4)	23 (3.1)	2 (0.3)	148 (19.7)^c^
14. Have you ever sent a “friend request“ to a patient or a member of the patient’s family from a private profile on an SNS?^d^	699 (92.8)^c^	33 (4.4)	3 (0.4)	18 (2.4)	N/A^e^

^a^SNS: social networking site.

^b^Item represents professional behavior on SNS.

^c^Response represents professional behavior on SNS.

^d^The options were “no,” “yes, to a patient,” “yes, to a family member,” and “yes, both,” respectively.

^e^N/A: not applicable.

The answer “I have never been in a situation where this could happen” is not a missing value, but it carries a conceptual meaning that must be distinguished from the answer “Never.” The assessment of whether that answer is professional or unprofessional depends on the content and direction of the item. Respondents who have never engaged in unprofessional behavior are professional, but so are those who never had an opportunity to act unprofessionally. Respondents who often practice behaviors on items marked with “b” are professional, and so are those who have never been in a situation to practice these behaviors because they have not been in a situation to behave unprofessionally.

For example, in the case of positive items (those representing professional behavior), such as “I asked a colleague’s permission to mention him/her in the post,” professional behavior is defined as a situation where the individual has never violated this rule because they have never mentioned colleagues in their posts or seek permission each time they mention them. Any other frequency level implies that, at some point, the person has posted about colleagues without their consent, which constitutes unprofessional behavior on SNS.

It is crucial here to differentiate between the absence of behavior of interest (requesting permission from colleagues when mentioning them in posts) in situations where it should have been sought (if mentioning them in posts) from the situations where it should not have been sought (because they never mention colleagues).

By contrast, for negative items (those representing unprofessional behavior), such as “I shared some information about the patient that I received through SNS with other people,” professional behavior is defined as situations where the individual has never engaged in such behavior or has not even been in a situation where they could engage in such behavior (eg, they do not communicate with patients via SNS, so they cannot receive patient information through this channel).

Therefore, the context of the absence of specific behaviors plays a pivotal role in distinguishing between professional and unprofessional behaviors. It is essential to combine the response “I have never been in a situation where this could happen” with the level of behavior frequency.

To construct the index, the frequency of behavior on each indicator was not graded but only considered as a binary value (professional vs unprofessional).

For items that measure unprofessional behavior, any degree of frequency other than “never” was considered unprofessional behavior. For items that measure professional behavior (eg, asking a colleague’s permission to mention them in a post), all those who did this never, rarely, or occasionally were considered unprofessional on that indicator, because this is the behavior they are expected to do always (or often in our scale).

### The Validation Process of the e-Professionalism Index—The Danger Aspect of SNSs

After specifying the scope and defining the indicators, the third step, according to Diamantopoulos and Winklhofer [28], refers to checking the collinearity of the indicators. Intercorrelations of the items in the e-professionalism instrument—the danger aspect of SNSs are shown in Multimedia Appendix 4. Given that these are binary variables, phi coefficients of associations were used. The correlation between the variables “On SNS, I choose with which patients I will make contact with and which I will not.” and “From a private profile, I communicate with patients regarding medical/dental problems and treatment.” (*r*=0.568) represents a moderate correlation and evokes the need to investigate potential multicollinearity. This suggests that those who communicated with patients via SNSs also chose with whom (patients) they would establish communication. As a formative approach is used, special care is needed before excluding indicators to preserve the instrument’s validity. Therefore, the VIF and MIMIC model were calculated. Multicollinearity was tested using a VIF with an additive index of e-professionalism, an aspect of the danger of SNS that was constructed as the sum of values on binary indicators. According to the conservative threshold [37], VIF values on all indicators were below the value of 2.5, which suggests that multicollinearity is not an issue.

The MIMIC model was implemented to check the external validity of the instrument. The path diagram of the MIMIC model is shown in Multimedia Appendix 5. Variables x_1_-x_14_ correspond to the items from Table 2. Items y_1_ (Communication with a patient through social media can be achieved without compromising doctor-patient confidentiality) and y_2_ (Social media have the potential to improve communication between a doctor and a patient) were chosen as reflective indicators.

The model showed good fit (*χ*^2^_13_=9.4, *P*=.742; *χ*^2^/*df* =.723; root-mean-square error of approximation<0.001; goodness-of-fit index=0.998; comparative fit index=1.000). However, 7 of the 14 items (x_1_, x_2_, x_3_, x_6_, x_7_, x_8_, and x_13_) did not have significant regression coefficients (γ) that can also be interpreted as validity coefficients [28]. The probable reason is that the measured reflective indicators did not measure the same domains as e-professional behavior; instead, they measured an attitude toward e-professionalism. Both items 11 (*P*<.001) and 12 (*P*=.02), which were investigated as potential problems of multicollinearity, have significant validity coefficients. Considering that, as well as an acceptable VIF, they were retained in the index to preserve the content validity to which formative models are particularly sensitive.

A higher value on the index means a higher degree of e-professionalism, that is, a lower incidence of unprofessional behavior on SNSs. The index results ranged from 0 to 14, and the average value in our sample was 10.60 (SD 2.173). The distribution of the index was skewed toward higher values (α_3_=–.44, *P*=.09), that is, toward the professional behavior of our respondents on SNSs.

The external validity of the index is supported by the correlation with other measured constructs. There was a statistically significant negative correlation between the index of e-professionalism (aspects of the danger of SNSs) and the scale of attitude toward SNSs (*r*=–0.225, *P*<.001).

### The Construction of the e-Professionalism Index—The Opportunity Aspect of SNSs

The construction of the e-professionalism index—the opportunity aspect of SNSs follows the same validation steps as the aspect of the dangers of SNSs [28].

E-professional behaviors described in the normative framework were operationalized into an instrument for measuring e-professionalism through the opportunity aspect of SNSs. The instrument contains 2 domains, measured by 6 items. All items are formulated in the same direction so a higher frequency measures a higher level of e-professionalism (Table 3).

**Table 3:** Domains, indicators, and items for the instrument of e-professionalism—opportunity aspect of SNSs^a^.

Domain and indicator		Item	Direction^b^
**Proactive posting of expert information of public health interest**			
	Sharing posts that contain general medical advice	I share posts on social media that contain general medical/dental advice.	+
	Sharing new scientific knowledge in the field of medicine	I use my profile to share information about new scientific knowledge in the field of medicine/dental medicine.	+
	Debunking medical myths and misinformation	I debunk medical/dental myths and misinformation by posting on SNS.	+
	Calling for public health actions	I use SNS to raise public awareness of public health actions.	+
	Encouraging responsible behavior	I create posts on SNS that call for responsible health behavior.	+
**Scientific objectivity**			
	Emphasis on distinguishing personal medical opinions from facts	In the posts, I clearly separate my personal opinion on a medical/dental issue from scientifically confirmed facts.	+

^a^SNS: social networking site.

^b^All items are formulated in the same direction so a higher frequency measures a higher level of e-professionalism.

The descriptive results for the items that measure the opportunity aspect of SNSs are shown in Table 4. While measuring the danger aspect of SNSs focused on occurrence, not on the frequency of occurrence, the frequency of each behavior is relevant with this instrument. All behaviors in this instrument have the characteristic of being desirable, but the absence of such behaviors is not unprofessional. If an MD or DMD practices these behaviors, they use opportunities of SNSs and contribute to their professionalism. However, if they do not practice any of these behaviors, or have never been in a situation where they can behave like that, it is not unprofessional, but misses the opportunity to use the advantages of SNSs.

**Table 4:** E-professionalism—opportunity aspect of SNSs^a^—descriptives (N=753).

	Never, n (%)	Rarely, n (%)	Occasionally, n (%)	Often, n (%)	I have never been in a situation where this could happen, n (%)
1. I debunk medical/dental myths and misinformation by posting on SNS.	355 (47.1)	130 (17.3)	87 (11.6)	16 (2.1)	165 (21.9)
2. I share posts on social media that contain general medical/dental advice.	312 (41.4)	167 (22.2)	128 (17.0)	28 (3.7)	118 (15.7)
3. I use SNS to raise public awareness of public health actions.	185 (24.6)	191 (25.4)	224 (29.7)	84 (11.2)	69 (9.2)
4. I use my profile to share information about new scientific knowledge in the field of medicine/dental medicine.	248 (32.9)	183 (24.3)	184 (24.4)	54 (7.2)	84 (11.2)
5. I create posts on SNS that call for responsible health behavior.	186 (24.7)	196 (26.0)	212 (28.2)	81 (10.8)	78 (10.4)
6. In the posts, I clearly separate my personal opinion on a medical/dental issue from scientifically confirmed facts.	170 (22.6)	49 (6.5)	77 (10.2)	149 (19.8)	371 (49.3)

^a^SNS: social networking site.

To construct an index reflecting the degree of e-professionalism in utilizing social networking opportunities, responses marked as “Never” or “I have never been in a situation where this could happen” are not considered contributions to e-professionalism and are coded as 0. Conversely, responses categorized as “Rarely,” “Occasionally,” and “Often” contribute to e-professionalism, representing 3 levels of engagement with the benefits of social networks and are coded as 1, 2, and 3 respectively.

### The Validation Process of the e-Professionalism Index—Opportunity Aspect of SNSs

The correlations between the items that constitute this index have higher values than those in the aspect of dangers of the SNS index (Multimedia Appendix 6). The item “I create posts on SNS that call for responsible health behavior” moderately correlates with several items (from *r*=0.418 to 0.714). To check if multicollinearity is present in this instrument, paying attention to the VIF is necessary.

VIF was calculated with an additive index of e-professionalism—opportunity aspect of SNSs. VIF values on all indicators are below the value of 2.5, which suggests no risk of multicollinearity, even according to a conservative interpretation.

Before excluding the item “I create posts on SNS that call for responsible health behavior,” an MIMIC model was created with all the items included, and a second model without that item was created to check for any changes in the model fit. The diagram of the MIMIC model is shown in Multimedia Appendix 5. Variables x_1_-x_6_ correspond to the items from Table 4. Items y_3_ (As MD/DMD, it is my duty to keep abreast of current trends in the use of SNS) and y_4_ (Guiding patients to online information is a new responsibility of MDs/DMDs in the digital age) were chosen as 2 reflective indicators.

The MIMIC model with all 6 items showed good fit characteristics (*χ*^2^_5_=2.880, *P*=.718; *χ*^2^/*df* = 0.576; root-mean-square error of approximation<.001; goodness-of-fit index=0.999; comparative fit index=1.000). However, 3 items (x_1_, x_3_, and x_5_) did not have significant regression coefficients (γ; *P*=.14, *P*=.44, and *P*=.19, respectively).

Considering the high correlations with other items, the VIF value that exceeds the limit of 2.5, and the regression coefficient γ that is not statistically significant (*P*=.19), item x_5_ was excluded from the e-professionalism index—opportunity aspect of SNSs. After excluding item x_5_, the fit of the MIMIC model did not change significantly (Δ*χ*^2^_1_=0.336, *P*=.56) and the fit of the model was *χ*^2^_4_=2.544, *P*=.718; *χ*^2^/*df* = 0.637; root-mean-square error of approximation<.001; goodness-of-fit index=0.999; comparative fit index=1.000.

The index of e-professionalism—opportunity aspect of SNSs was created as the sum of the values of the remaining 5 recoded variables. A higher value on the e-professionalism index means a higher degree of e-professionalism. The index results ranged from 0 to 15 (mean 4.13, SD 3.712). The distribution of the index was skewed toward lower values (α_3_=0.67, *P*=.09), showing that 24% (181/753) of respondents do not take advantage of SNSs at all.

The external validity of the index of e-professionalism—opportunity aspect of SNSs is supported by the correlation with other measured constructs. There was a statistically significant positive correlation between the index and the scale of attitude toward SNSs (*r*=0.338, *P*<.001).

## Discussion

### Principal Findings

As far as the authors are aware, this is the first measure constructed to measure the e-professional behavior of MDs and DMDs, with the created indexes of opportunity and the danger aspects of SNSs being the first attempt at using a formative approach in the research of professionalism in general and in e-professionalism. The final instrument for measuring the e-professional behavior of MDs and DMDs consists of 19 items that form 2 indexes. Index of e-professionalism—the danger aspect of SNS, which is formed by 14 items, and the index of e-professionalism—opportunity aspect of SNS, which is formed by 5 items.

These novel indexes can be used to measure the level of e-professional behavior among MDs and DMDs, which can have potential real-world applications. The main implications can be utilized in education for young medical and dental professionals and the development of guidelines for improving e-professionalism. If the instrument were applied on a representative sample, it could yield valuable data to enable the implementation of data-based policies with specific behaviors of interest. Investigation of the external validity of both e-professionalisms showed acceptable results. There was a statistically significant negative correlation between the index of e-professionalism—the danger aspect of SNSs and the scale of attitude toward SNSs (*r*=–0.225, *P*<.001). This is the theoretically expected direction of the correlation because the more positive attitude the respondents have about SNSs, the more inclined they are to use them when working with patients, which according to the normative framework, represents unprofessional behavior. The statistically significant positive correlation between the index of e-professionalism—opportunity aspect of SNSs and the scale of attitude toward SNSs (*r*=0.338, *P*<.001) is also theoretically expected because the more positive attitude toward SNSs doctors have, the more likely they will take advantage of the benefits of SNSs.

In the index of e-professionalism—the danger aspect of SNSs, all initially operationalized indicators were retained. In the index of e-professionalism—the opportunity aspect of SNSs, item x_5_ (I create posts on SNS that call for responsible health behavior) measuring the indicator “Encouraging responsible behavior” was excluded. The formative approach suggests cautious consideration of managing the content validity of the model. It seems that respondents understood item x_5_ very similarly to item x_3_ (I use SNS to raise public awareness of public health actions.). After testing the indicators in the MIMIC model, the authors concurred that the content validity is not threatened by excluding this item, and multicollinearity would pose a more significant problem than losing a very subtle difference in the contents of these items.

### Comparison With Prior Work

Conceptual domains recognized in this study only partially overlap with domains in the instrument of (offline) professional behavior [19] and the instrument for measuring attitudes toward e-professionalism [35]. Kelley et al [19] recognized a domain called “Upholding principles of integrity and respect,” which corresponds to the domain “Confidentiality” in this study, as well as “Citizenship and professional engagement” [19], which corresponds to “Proactive posting of expert information of public health interest.” In an instrument for measuring attitudes toward e-professionalism, Marelić et al [35] recognized the domain “Ethical aspects” that theoretically includes HIPAA violations and therefore corresponds to the domain “Confidentiality” in this study, and the domain “Physicians in the digital age” that corresponds to “Contact with patients”. However, the instrument of (offline) professional behavior contains domains that are not comparable to e-professional behavior, and the instrument for measuring attitudes toward e-professionalism contains domains that are not applicable for behavior measurement, and because of potential cognitive dissonance, measuring attitude is not a replacement for behavior measurement.

### Limitations

The first limitation of this study is the low response rate (1013/23,178, 4.37%). Previous research has indicated that these professions have low survey response rates, especially in e-mailing surveys using web-based formats [38-42]. Time, confidentiality concerns, and topic relevance are some of the main reasons for their low survey participation [40]. Previous research has indicated that declining response rates among HCPs may be attributed to various factors, including heightened requests to participate in surveys and increased workloads. This increase in workload encompasses both the rising number of patients and administrative responsibilities [38,39].

One factor likely contributing to the low response rate in this study is the demanding schedule of MDs and DMDs. The estimated time required to complete our survey was lengthy, ranging from 10 to 15 minutes, due to the inclusion of a complex and comprehensive questionnaire containing 40 questions. Moreover, the survey was conducted during the COVID-19 pandemic (February to July 2021), a period marked by heightened strain on the health care system. MDs, especially those in Croatia, were confronted with extreme workloads and specific working conditions during this time. Additionally, MDs received numerous invitations to participate in web-based surveys, particularly regarding the impact of the COVID-19 pandemic on their physical or mental health. Given these circumstances, our study’s focus on e-professionalism may have been perceived as of lower interest, potentially further reducing doctors’ willingness to participate in research.

However, our objective in creating and validating new indexes did not prioritize achieving representativeness in our sample or generalizing our findings to the entire population of MDs and DMDs in Croatia. Instead, our focus was on assessing the suitability of the developed measurement instruments across various medical professions, using a nonprobabilistic purposive sample. Our final sample comprised responses obtained from the population of interest for this study, specifically MDs and DMDs who use at least one SNS. It is worth noting that the number of responses received in our survey (507 MDs and 246 DMDs) exceeded the initially planned sample size (140 MDs and 140 DMDs) by a considerable margin.

The second limitation concerns a relatively large proportion of respondents (ranging from 69/753, 9.2%, to 371/753, 49.3%) who selected the option “I have never been in a situation where this could happen” for certain items. It remains unclear why they did not simply respond with “Never.” The reasons behind this choice are ambiguous. It is possible that some respondents are passive users of SNSs, thus not engaging in any content publication and consequently unable to exhibit unprofessional behavior. Alternatively, it could be that these respondents do not work directly with patients, rendering items related to violations of the HIPAA irrelevant to them. Another possibility is that they perceive their standards of professionalism to be exceptionally high, leading them to believe they would never engage in such behavior. While this issue does not affect the measurement of the occurrence of e-(un)professional behavior, it does impede a detailed understanding of the frequency of e-unprofessional behavior. Addressing this limitation could be a focus of future research and modifications to the measurement instrument, but this should be preceded by gaining new insights into the e-professional behaviors of MDs and DMDs.

The third limitation involves the potential for bias associated with using a self-reporting approach to measurement. Similar to other self-report measures in medicine, 2 key biases often arise: recall bias and social desirability bias [43]. Recall bias in our study could be attributed to the lack of a specified timeframe, such as “during the last year.” We chose this approach because it represents the initial assessment of such behaviors, and we faced a scarcity of existing data on this subject. Introducing a specific timeframe in future research could aid in mitigating potential recall bias. The potential for social desirability bias stems from 2 sources. First, the nature of the measurement itself requires HCP respondents to self-report potentially unprofessional behaviors, including some that may constitute violations of HIPAA. The other factor to consider is that respondents were contacted to participate in our research through the same institutions responsible for granting and revoking licenses to practice medicine/dental medicine. Despite our assurance of anonymity in the study, respondents may have felt compelled to provide socially desirable answers on certain items. One method to mitigate or control social desirability bias is to include positive items, such as those measuring professional behaviors, alongside other items. An additional approach to address both biases, which could serve as a recommendation for future research, involves further refinement and validation of the instrument. This could be achieved by comparing self-reported data with information obtained through web scraping of respondents’ SNS profiles, particularly focusing on visible behaviors.

The fourth limitation arises from the potential mismatch between the use of reflective indicators y_1_-y_4_ in the MIMIC model and the nature of the created indexes, which are intended to measure e-professionalism as behavior. However, the reflective variables used in the model measure attitude. While this approach was necessary for creating the MIMIC model in this study, there is a possibility that cognitive dissonance [4,21] may compromise the fit of the model.

The fifth limitation to note is that the sources used to establish a normative framework were relevant to the time and location of this research. However, their applicability to other countries and populations of HCPs, or their accuracy over time, may be limited. For example, the ABIM e-professional conduct guidelines [5] are relatively dated, and while they represent fundamental values of professionalism, they may not fully encompass changes in societal values that have occurred since the emergence of SNSs. Specific behaviors measured in these indexes may require revision or supplementation in the future. Moreover, additional studies conducted after the development of this index may offer new insights into creating a normative framework for defining e-professional behaviors [44].

### Future Directions

In considering avenues for enhancing both the instruments used in this study and future research directions, it becomes apparent that there are opportunities for improvement and deeper exploration. One potential extension of this study, which could lead to a more thorough understanding of the topic, involves testing the indexes on specific subsamples, particularly within specialties such as dermatology and reconstructive and cosmetic surgery. These specialties may involve visual representations of procedures, such as “before and after” images [34], which could pose potential threats to e-professionalism.

Improving the quality of external validity assessment can be achieved by incorporating self-evaluation of e-professionalism into the MIMIC model. This addition would enhance the content validity of the model by supplementing existing reflective indicators used in the research. Furthermore, self-evaluation of e-professionalism would serve as a valuable tool for evaluating the nomological network of the instrument. It would provide insights into the direction and strength of correlation among individual indicators of e-professionalism, the e-professionalism indices themselves, and potential predictors for model creation.

Future attempts aimed at measuring e-professionalism could focus on investigating the underlying reasons behind responses such as “I have never been in a situation where this could happen." It is plausible that a more precise definition of items or the inclusion of specific examples could serve as mechanisms to help respondents differentiate between behaviors they never engage in and those they may never encounter. By refining the clarity and specificity of survey items, researchers can facilitate a more accurate assessment of respondents’ experiences and perceptions related to e-professional behavior. This approach could lead to a deeper understanding of the nuances involved in professional conduct within the context of SNSs.

### Conclusions

In this paper, an instrument for measuring the e-professional behavior of MDs and DMDs was developed and validated using the formative approach. Following the validation process, the instrument comprises 19 items, which contribute to the formation of 2 indexes. The first index, focusing on the danger aspect of SNSs, is composed of 14 items that were dichotomized before index construction. The second index, which examines the opportunity aspect of SNSs, is composed of 5 items that were recoded as 4-point items before index construction.

These innovative indexes offer a means to gauge the level of e-professional behavior among MDs and DMDs. This marks the first measure specifically designed to assess the e-professional behavior of MDs and DMDs. The paper demonstrates the feasibility of investigating e-professional behavior using a formative approach, representing an advancement over existing measuring instruments. This approach provides a means to mitigate the impact of cognitive dissonance between attitudes and the actual behavior of MDs and DMDs.

The validation process confirmed that these indexes serve as a robust measure of e-professional behavior. Nevertheless, the instrument has been scrutinized for potential areas of enhancement, and suggestions for improvements have been proposed for future iterations of the instrument.

## Appendix

Multimedia Appendix 1 Checklist for Reporting Results of Internet E-Surveys (CHERRIES).

Multimedia Appendix 2 Descriptive characteristics of reflective indicators for the MIMIC models of e-professionalism (N=753).

Multimedia Appendix 3 Type of workplace and specialization status of the respondents.

Multimedia Appendix 4 Intercorrelations of items in the e-professionalism instrument - danger aspect of SNSs (N=753).

Multimedia Appendix 5 MIMIC e-Professionalism models.

Multimedia Appendix 6 Intercorrelations of items in the e-professionalism instrument - opportunity aspect of SNSs (N=753).

## Abbreviations

ABIM: The American Board of Internal Medicine
CCDM: Croatian Chamber of Dental Medicine
CHERRIES: Checklist for Reporting Results of Internet E-Surveys
CHIF: Croatian Health Insurance Fund
CMC: Croatian Medical Chamber
DMD: doctor of dental medicine
HCP: health care professional
HIPAA: Health Insurance Portability and Accountability Act
MD: doctor of medicine
MIMIC: multiple indicators multiple causes
SNS: social networking site
VIF: variance inflation factor

## Data Availability

The data sets used or analyzed during this study are available from the corresponding author on reasonable request.
