# Supplementary material for: Measuring e-Professional Behavior of Doctors of Medicine and Dental Medicine on Social Networking Sites: Indexes Construction With Formative Indicators
Source: JMIR Med Educ. 2024 Feb 27;10:e50156. doi: 10.2196/50156 (PMC10933720; doi:10.2196/50156)
Supplement: Multimedia Appendix 1 [file mededu_v10i1e50156_app1.docx]

**MULTIMEDIA APPENDIX 1**. Checklist for Reporting Results of Internet E-Surveys (CHERRIES).

| **Item category** | **Checklist item** | **Explanation** |
| --- | --- | --- |
| **Design** |  |  |
|  | Describe survey design | The target population of this survey were doctors of medicine (MD) and dental medicine (DMD) in Croatia. The sampling frame includes MD members of the Croatian Medical Chamber (CMC) and DMD members of the Croatian Chamber of Dental Medicine (CCDM), who use at least one Social Networking Site.  The type of sample was a non-probabilistic purposive sample. |
| **IRB** |  |  |
|  | IRB approval | Both the study and the questionnaire were approved by the ethical boards of the University of Zagreb School of Medicine (641-01/18-02/01) and the University of Zagreb School of Dental Medicine (05-PA-24-2/2018).  Formal approval was obtained from the governing bodies of both the CMC and CCDM for the use of the complete mailing lists of MDs and DMDs who are members of the CMC (900-06/20-01/11) and CCDM (900-01/21-01/02). |
|  | Informed consent | The mailing lists used to distribute the survey were the official full membership emailing lists of the CMC and CCDM.  The email included a brief text about the study’s objective, the expected time to complete the survey, and the person and university responsible for conducting the study.  After clicking on the survey link, before the first question, there was a detailed description of the purpose of the study, which data will be stored, who the research team is and the purpose of the study.  The respondents had to click informed consent to start filling out the questionnaire. |
|  | Data protection | No identification data was collected. |
| **Development and pre-testing** |  |  |
|  | Development and testing | This paper describes the design and validation process of the new instrument for measuring the e-professional behavior of MDs and DMDs.  The functionality of the electronic questionnaire has been tested by the researchers before fielding the questionnaire. |
| **Recruitment process and description of the sample having access to the questionnaire** |  |  |
|  | Open survey versus closed survey | This was a closed survey. Only members of CMC/CCDM received an email with the survey link. |
|  | Contact mode | The mailing lists used to distribute the survey were the official full membership emailing lists of the CMC and CCDM. |
|  | Advertising the survey | The members of the SMePROF research team provided the CMC and CCDM with email text, which the respective chambers forwarded to their members. The link to the survey was embedded in the email invitation to participate. |
| **Survey administration** |  |  |
|  | Web/E-mail | The link to the Microsoft Forms questionnaire was sent through e-mail. The responses were entered automatically into the database by the Microsoft Forms platform. |
|  | Context | The members of the CMC/CCDM are respectively MDs and DMDs. Membership in the chamber is necessary to obtain a license for practicing medicine/dental medicine in Croatia.  The cambers sent email invitations with the survey link, however, not all members are included in the mailing list. At the time of the survey, the CMC’s emailing list contained 15,562 email addresses of MDs, and the CCDM’s email list contained 7,616 email addresses of DMDs. |
|  | Mandatory/voluntary | The survey was voluntary. |
|  | Incentives | None. |
|  | Time/Date | From February 2021 to July 2021 |
|  | Randomization of items or questionnaires | None. |
|  | Adaptive questioning | Adaptive questioning was used in the questionnaire but not for the instrument used in this paper. |
|  | Number of Items | The new instrument for measuring the e-professional behavior of MDs and DMDs, presented in this study, is part of a more extensive questionnaire called SMePROF-D, developed within the project „Dangers and benefits of social networks: E-professionalism of healthcare professionals.” The new instrument contains 20 items used to create two e-professionalism indexes. To assess the external validity of the instrument, an additional 16 items are used. Six items are used to measure the sociodemographic characteristics of the sample. The total number of items used in this paper is 42. |
|  | Number of screens (pages) | Microsoft Forms uses a “long format” with adaptive questioning, which means that the number of screens varies greatly depending on answers and the device used for filling out the form, preventing us from precisely determining the number of screens in this instrument. However, for instance, on a screen with a resolution of 1920 x 1080 pixels, the total number of screens required to display all 42 items in this study is 12 |
|  | Completeness check | The settings in Microsoft Forms were set so that only completed surveys could be submitted. Therefore, no completeness check was required. |
|  | Review step | Respondents could change their answers at any time before submitting the response. There was no Review step before finishing the questionnaire. |
| **Response rates** |  |  |
|  | Unique site visitor | N/A |
|  | View rate (Ratio of unique survey visitors/unique site visitors) | N/A |
|  | Participation rate (Ratio of unique visitors who agreed to participate/unique first survey page visitors) | The participation rate was not calculated. |
|  | Completion rate (Ratio of users who finished the survey/users who agreed to participate) | Because of the Microsoft Forms settings, the users who did not finish the survey were not included in the sample, so the completion rate is not applicable to this research. |
| **Preventing multiple entries from the same individual** |  |  |
|  | Cookies used | Cookies were not used. |
|  | IP check | IP addresses were not collected. |
|  | Log file analysis | IBM SPSS statistics 26 feature “Identify duplicate cases” was used on the completed dataset.  Four cases were recognized as duplicates with identical responses on 45 variables and excluded from the analysis. |
|  | Registration | N/A |
| **Analysis** |  |  |
|  | Handling of incomplete questionnaires | Only complete surveys were analyzed. Incomplete surveys were not collected. |
|  | Questionnaires submitted with an atypical timestamp | N/A |
|  | Statistical correction | N/A |
