# Supplementary material for: Measuring e-Professional Behavior of Doctors of Medicine and Dental Medicine on Social Networking Sites: Indexes Construction With Formative Indicators
Source: JMIR Med Educ. 2024 Feb 27;10:e50156. doi: 10.2196/50156 (PMC10933720; doi:10.2196/50156)
Supplement: Multimedia Appendix 2 [file mededu_v10i1e50156_app2.doc]

**MULTIMEDIA APPENDIX 2**. Descriptive characteristics of reflective indicators for the MIMIC models of e-professionalism (N=753).

| Item | I completely disagree | I disagree | I don't know, I'm not sure | I agree | I completely agree |
| --- | --- | --- | --- | --- | --- |
|  | n (%) | n (%) | n (%) | n (%) | n (%) |
| y_1_ Communication with a patient through social media can be achieved without compromising doctor-patient confidentiality | 40 (5.3) | 138 (18.3) | 240 (31.9) | 299 (39.7) | 36 (4.8) |
| y_2_ Social media have the potential to improve communication between a doctor and a patient | 39 (5.2) | 117 (15.5) | 238 (31.6) | 336 (44.6) | 23 (3.1) |
| y_3_ As MD/DMD, it is my duty to keep abreast of current trends in the use of SNS. | 67 (8.9) | 134 (17.8) | 174 (23.1) | 322 (42.8) | 56 (7.4) |
| y_4_ Guiding patients to online information is a new responsibility of MDs/DMDs in the digital age | 121 (16.1) | 258 (34.3) | 221 (29.3) | 138 (18.3) | 15 (2.0) |
