# Supplementary material for: Measuring e-Professional Behavior of Doctors of Medicine and Dental Medicine on Social Networking Sites: Indexes Construction With Formative Indicators
Source: JMIR Med Educ. 2024 Feb 27;10:e50156. doi: 10.2196/50156 (PMC10933720; doi:10.2196/50156)
Supplement: Multimedia Appendix 3 [file mededu_v10i1e50156_app3.docx]

**MULTIMEDIA APPENDIX 3**. Type of workplace and specialization status of the respondents.

|  | MD (n=507) | DMD (n=246) | Total (N=753) |
| --- | --- | --- | --- |
|  | n (%) | n (%) | n (%) |
|  |  |  |  |
| **Type of workplace** |  |  |  |
| Public health institution | 356 (70.2) | 56 (22.8) | 412 (54.7) |
| Private health institution / without a contract with CHIF | 39 (7.7) | 74 (30.1) | 113 (15) |
| Faculty | 12 (2.4) | 11 (4.5) | 23 (3.1) |
| A private institution with a contract with CHIF | 58 (11.4) | 90 (36.6) | 148 (19.6) |
| A combination of a faculty and a health institution | 21 (4.1) | 7 (2.8) | 28 (3.7) |
| Other | 21 (4.1) | 8 (3.2) | 29 (3.8) |
|  |  |  |  |
| **Specialization status** |  |  |  |
| Without specialization | 104 (20.5) | 182 (73.9) | 286 (38) |
| Resident | 205 (40.4) | 20 (8.1) | 225 (29.9) |
| Specialist | 198(39.1) | 44 (17.9) | 242 (32.1) |
