# Supplementary material for: Measuring e-Professional Behavior of Doctors of Medicine and Dental Medicine on Social Networking Sites: Indexes Construction With Formative Indicators
Source: JMIR Med Educ. 2024 Feb 27;10:e50156. doi: 10.2196/50156 (PMC10933720; doi:10.2196/50156)
Supplement: Multimedia Appendix 4 [file mededu_v10i1e50156_app4.docx]

**MULTIMEDIA APPENDIX 4**. Intercorrelations of items in the e-professionalism instrument - danger aspect of SNSs (N=753).

| Recoded (binomial) item^a b^ | 1 | 2 | 3 | 4 | 5 | 6 | 7 |
| --- | --- | --- | --- | --- | --- | --- | --- |
| 1. Not asking a colleague's permission to mention them in a post. | 1 |  |  |  |  |  |  |
| 1. Not controlling the visibility of posts appropriate to the content. | 0.270** | 1 |  |  |  |  |  |
| 1. Not controlling the visibility of content posted by others | 0.229** | 0.263** | 1 |  |  |  |  |
| 1. Not paying attention to maintaining professionalism in the posts. | 0.186** | 0.169** | 0.158** | 1 |  |  |  |
| 1. Sharing patient information obtained through SNS with other persons. | 0.100** | 0.110** | 0.074* | 0.039 | 1 |  |  |
| 1. Posting a photo of the patient without their knowledge. | 0.078* | 0.067 | 0.034 | -0.064 | 0.149** | 1 |  |
| 1. Inclusion of patient data collected through SNS in medical documentation without their knowledge. | 0.020 | -0.008 | 0.040 | 0.054 | 0.172** | 0.176** | 1 |
| 1. Sharing medical/dental advice on SNS without the name being visible. | 0.083* | 0.060 | 0.030 | 0.065 | 0.085* | 0.172** | 0.177** |
| 1. Posting content that shows informal situations in the workplace. | 0.134** | 0.130** | 0.063 | 0.064 | 0.168** | 0.100** | 0.037 |
| 1. Posting information about the patient. | 0.044 | 0.047 | 0.026 | 0.006 | 0.182** | 0.205** | 0.239** |
| 1. Communicating with patients regarding medical/dental problems and treatment from a private profile. | 0.146** | 0.065 | 0.051 | -0.042 | 0.153** | 0.181** | 0.058 |
| 1. Selecting patients on SNS with whom communication is established. | 0.122** | 0.049 | 0.026 | -0.069 | 0.130** | 0.127** | 0.057 |
| 1. Sending a friend request to a patient or a member of the patient's family from a private profile | 0.076* | 0.076* | 0.072* | -0.022 | 0.120** | 0.100** | 0.104** |
| 1. Use of profanity or vulgar expressions in posts. | 0.122** | 0.143** | 0.039 | 0.305** | 0.159** | 0.079* | 0.058 |

^a^ phi-coefficients of association were used
^b^**P*<0.05; ***P*<0.01

**MULTIMEDIA APPENDIX 4**. Intercorrelations of items in the e-professionalism instrument - danger aspect of SNSs (N=753). (continued)

| Recoded (binomial) item^a b^ | 8 | 9 | 10 | 11 | 12 | 13 |
| --- | --- | --- | --- | --- | --- | --- |
| 1. Sharing medical/dental advice on SNS without the name being visible. | 1 |  |  |  |  |  |
| 1. Posting content that shows informal situations in the workplace. | 0.072* | 1 |  |  |  |  |
| 1. Posting information about the patient. | 0.143** | 0.128** | 1 |  |  |  |
| 1. Communicating with patients regarding medical/dental problems and treatment from a private profile. | 0.087* | 0.251** | 0.106** | 1 |  |  |
| 1. Selecting patients on SNS with whom communication is established. | 0.072* | 0.252** | 0.164** | 0.568** | 1 |  |
| 1. Sending a friend request to a patient or a member of the patient's family from a private profile | 0.114** | 0.165** | 0.105** | 0.308** | 0.303** | 1 |
| 1. Use of profanity or vulgar expressions in posts. | 0.088* | 0.154** | 0.073* | 0.024 | 0.044 | 0.044 |

^a^ phi-coefficients of association were used
^b^**P*<0.05; ***P*<0.01
