# Supplementary material for: Measuring e-Professional Behavior of Doctors of Medicine and Dental Medicine on Social Networking Sites: Indexes Construction With Formative Indicators
Source: JMIR Med Educ. 2024 Feb 27;10:e50156. doi: 10.2196/50156 (PMC10933720; doi:10.2196/50156)
Supplement: Multimedia Appendix 5 [file mededu_v10i1e50156_app5.docx]

**MULTIMEDIA APPENDIX 5**. MIMIC e-Professionalism models.

**MIMIC e-Professionalism model – the danger aspect of SNSs (N=753).**


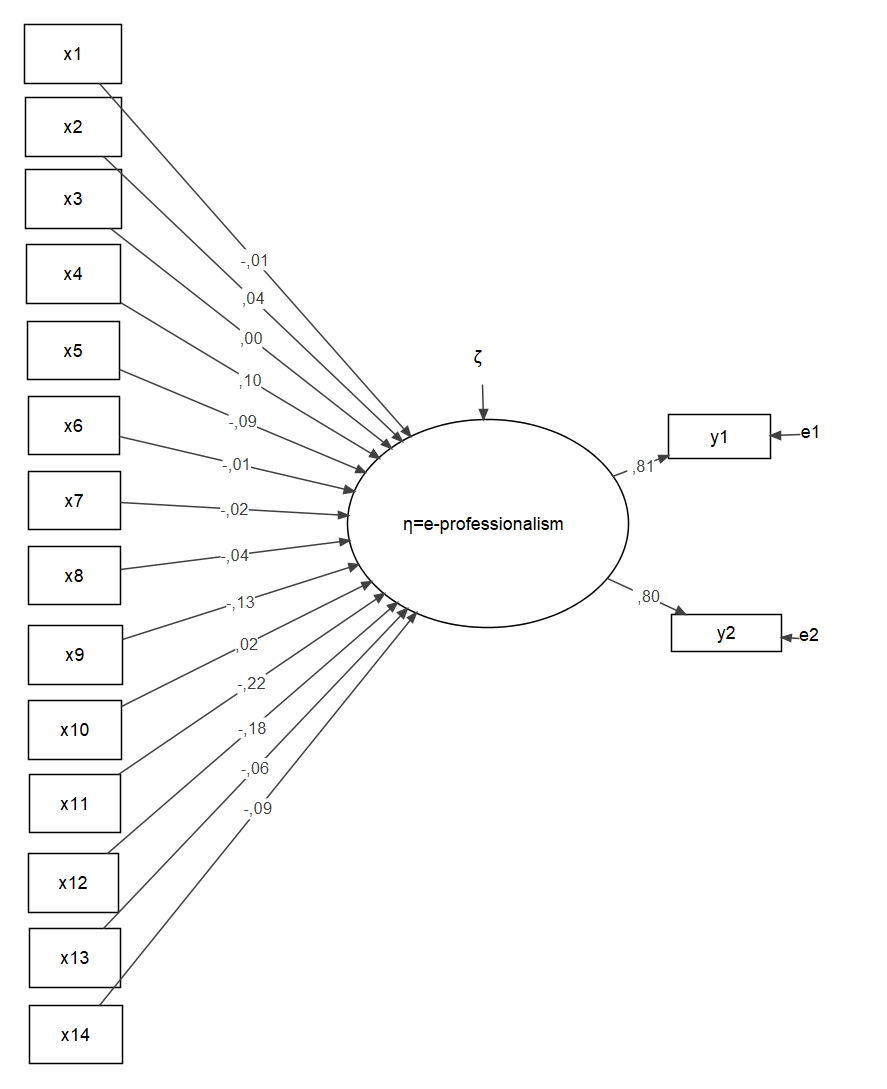


Correlations between the x variables and interaction labels are not shown for diagram clarity.

**MIMIC e-Professionalism model – opportunity aspect of SNSs (N=753).**


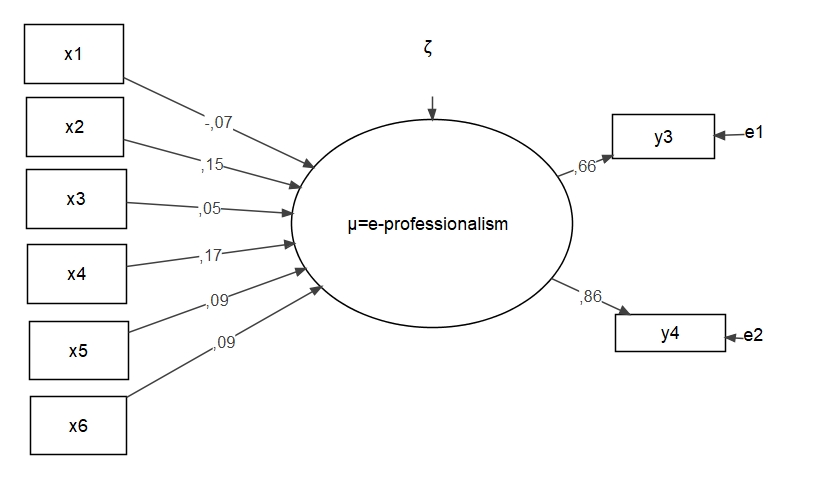


Correlations between the x variables and interaction labels are not shown for diagram clarity.
