# Supplementary material for: Measuring e-Professional Behavior of Doctors of Medicine and Dental Medicine on Social Networking Sites: Indexes Construction With Formative Indicators
Source: JMIR Med Educ. 2024 Feb 27;10:e50156. doi: 10.2196/50156 (PMC10933720; doi:10.2196/50156)
Supplement: Multimedia Appendix 6 [file mededu_v10i1e50156_app6.doc]

**MULTIMEDIA APPENDIX 6**. Intercorrelations of items in the e-professionalism instrument - opportunity aspect of SNSs (N=753).

| Recoded 4-point item^a b^ | 1 | 2 | 3 | 4 | 5 |
| --- | --- | --- | --- | --- | --- |
| 1. I debunk medical/dental myths and misinformation by posting on SNS. | 1 |  |  |  |  |
| 1. I share posts on social media that contain general medical/dental advice. | 0.583** | 1 |  |  |  |
| 1. I use SNS to raise public awareness of public health actions. | 0.399** | 0.578** | 1 |  |  |
| 1. I use my profile to share information about new scientific knowledge in the field of medicine/dental medicine. | 0.451** | 0.620** | 0.632** | 1 |  |
| 1. I create posts on SNS that call for responsible health behavior. | 0.418** | 0.604** | 0.751** | 0.714** | 1 |
| 1. In the posts, I clearly separate my personal opinion on a medical/dental issue from scientifically confirmed facts. | 0.350** | 0.371** | 0.364** | 0.379** | 0.421** |

^a^ Pearson's r correlation coefficient was used
^b^ **P*<0.05; ***P*<0.01
